# Eutrophication and the Ecological Health Risk

**DOI:** 10.3390/ijerph17176332

**Published:** 2020-08-31

**Authors:** Soon-Jin Hwang

**Affiliations:** Department of Environmental Health Science and Human and Eco-Care Center, Konkuk University, Seoul 05029, Korea; sjhwang@konkuk.ac.kr

**Keywords:** eutrophication, ecological health and risk, climatic change, harmful algal blooms, cyanobacteria, potential hazards, freshwater ecosystem

## Abstract

This Special Issue focuses on eutrophication and related ecological health risks—one of the biggest challenges to sustainable water management. It is increasingly recognized that eutrophication has multidimensional consequences for water quality, both ecosystem and human health, as well as economic activities. These consequences depend on site-specific conditions, specifically, the ecological stability of the system, land use types, climate change, and the presence of other contaminants, including infectious disease agents. This Special Issue contains ten research papers that focus on, among other factors, phosphorus, cyanobacteria, off-flavor substances, macroinvertebrates, chemical stress, and land-use effects, thereby increasing our understanding of the multidimensional effects of eutrophication.

## 1. Introduction

Freshwater is a crucial resource for the survival of life on earth [1,2]. However, global freshwater ecosystems are facing unprecedented threats due to anthropogenic activities, with climate change further aggravating these threats and related consequences in unpredictable ways [2].

Eutrophication is one of the most prevalent challenges faced by freshwater systems globally [3,4,5] and has a long history in terms of water management [6]. Eutrophication leads to major disruptions of aquatic ecosystems, affecting related goods and services [7], ecosystem and human health [8], as well as economic activities [9].

Within this context, harmful algal blooms represent one of the major ecological health risks associated with eutrophication [10]. This is especially true for cyanobacteria [11] that produce harmful materials, such as toxins [12,13] and off-flavor substances [14,15], which are potential hazards for both human and wildlife health [12,16]. Nutrient enrichment can interact with site-specific conditions, such as the presence of other contaminants, including infectious disease agents, thereby threatening the ecological stability of the system. Furthermore, the accelerated eutrophication of aquatic ecosystems has also been associated with the emergence of new diseases through both direct and indirect pathways [16], emphasizing the importance of acknowledging and understanding the mechanisms linking eutrophication and host–pathogen interactions. To ensure the sustainability of freshwater systems, a better understanding, as well as efficient assessment and control of eutrophication, is essential.

In summary, the Special Issue “Eutrophication and the Ecological Health Risk” aims to share results and findings from current research on eutrophication and related ecological health risks, with a special focus on freshwater sustainability.

### Current Special Issue

Notably, the studies included in this Special Issue reflect only a small and specific portion of this research field. Although only a few articles in this collection focus on the overall scope of the eutrophication and related ecological health risks, I would like to highlight the importance of broadening the context to include multidimensional problems when presenting results on this interesting and challenging subject.

Herein, I summarize the contributed papers in the context of eutrophication and related risks to the health of ecological systems to paint an overall picture of this Special Issue (Table 1). Three studies focused on “eutrophication” [17,18,19], four on “ecological health risk” [20,21,22,23], and a further three papers on “ecosystem health and services” [24,25,26] have been included in this issue. A more detailed summary of all articles is offered in the following section.

## 2. Contributions

The call for submissions to this Special Issue “Eutrophication and the Ecological Health Risk (EEHR)” stretched from December 2018 to March 2020. A total of 15 submissions were received, of which 10 were accepted for publication.

Kong et al. [17] evaluated the effect of phytoplankton-derived particulate organic matter (PPOM) on endogenous phosphorus (P) cycling and related impacts on cyanobacteria blooms, PPOM characteristics, degradation mechanisms, as well as the growth of P-deficient *Microcystis aeruginosa* in Lake Taihu, China. The authors used physical and chemical indexing and molecular particulate P analysis with ^31^P nuclear magnetic resonance to investigate alkaline phosphatase activity in particulate matter and water. Their results indicate PPOM to be the most important P-pool in water columns during cyanobacterial blooms. Furthermore, particulate orthophosphate was determined to be the main P-species released during the early stage of PPOM degradation. The authors highlight the contribution of P released during the degradation of PPOM for the control of lake eutrophication.

Wan et al. [18] used the soil Po fraction method to analyze the characteristics and distribution of organic phosphorus (Po) fractions in surface sediments of seven rivers that flow into Hongze Lake, China. In terms of the relative contribution of Po fractions in sediment, residual Po > HCl-Po > fulvic acid-Po > humic acid-Po > labile organic phosphorus. Organic phosphorus occurred primarily as moderately labile organic phosphorus in whole sediment. Significant correlations were reported between all Po forms and total phosphorus (TP), inorganic phosphorus (Pi), and Po. Furthermore, non-labile organic phosphorus showed the strongest correlation with TP. The results from this study provide a basis for the phosphorus cycle and a new perspective on eutrophication control of shallow lakes.

Zulkefli et al. [19] evaluated the effect of different biotic (bacterial activity) and abiotic factors (temperature, pH, light) on extracellular environmental DNA (exDNA) using 16S rDNA sequences extracted from both the sediment of a eutrophic lake and *Anabaena variabilis* cultured in the laboratory. Their results indicate that temperature, both independently and in combination with bacterial abundance, has a significant effect on the degradation of exDNA. In the study, the highest degradation rate was observed in samples exposed to high microbial activity, with exDNA degrading completely within a single day. However, light intensity and pH showed little effect on the degradation rate. The authors suggest that the degradation of exDNA in freshwater ecosystems is driven by the combination of both biotic and abiotic factors and can occur rapidly under specific conditions.

Kim et al. [20] carried out a methodological study to design a set of primers for detecting and quantifying 2-MIB-synthesizing cyanobacteria based on mibC gene sequences (encoding 2-MIB synthesis-catalyzing monoterpene cyclase) from various Oscillatoriales and Synechococcales cyanobacterial strains deposited in the GenBank. The designed mibC primers showed improved amplification efficiency and a high correlation between related variables. The primers are an efficient tool for identifying cyanobacterial strains possessing mibC genes and for evaluating mibC gene expression as an early warning of cyanobacterial blooms.

Ji et al. [21] investigated the behavioral response of an oligochaete, *Lumbriculus variegatus*, to copper sulfate, and demonstrated the effectiveness of using body segment data from these line-shaped animals in the detection of biological responses. Line body shape detection with parameters expressing body segments broadens the scope of behavioral characterization of elongated animals, adding an extra dimension that reveals the complexity of line-shaped bodies compared with point detection in round-shaped species. Notably, the patterns of time-series line movements of the test oligochaete were determined using a recurrent self-organizing map. 

Jo et al. [22] used a DNA meta-barcoding approach to identify the gut contents and analyze the feeding behavior of fourth-instar larvae of a chironomid (*Dicrotendipes fumidus*) that inhabits large-scale weirs. Their results indicate that the most common operational taxonomic unit (OTU) among individuals included phytoplankton, such as *Tetrahymena* sp., *Pseudopediastrum* sp., *Tetradesmus dimorphus*, *Biddulphia tridens*, *Desmodesmus armatus*, and other *Desmodesmus* spp. Calculations of the selectivity index (E’) show that chironomid larvae have a significant preference for certain phytoplankton taxa. Furthermore, results from this study suggest that combinations of different parameters, including physicochemical water quality, prey organism (small-sized single cell) particle size, and chemical effects (chemokinesis), influence the feeding behavior of Chironomidae larvae. The authors emphasize the potential of DNA meta-barcoding in the assessment of biological indicators and the impact on food-web structures in large-scale weirs using dietary insights.

Park et al. [23] evaluated the growth response and intracellular proline accumulation of a green alga, *Scenedesmus quadricauda*, isolated from brackish water, against dissolved salt stress due to N and P enrichment. Their results showed the highest salinity concentration to inhibit the growth rate of the test alga, regardless of specific nutrient levels. However, with an increase in nutrient enrichment, the alga showed increased tolerance to dissolved salts, due to intracellular proline synthesis. The study highlights the potential of nutrient enrichment in reducing the adverse effects of dissolved salt stress on the test alga by promoting cell growth and proline synthesis. Furthermore, results from the study suggest that the tolerance of algae to dissolved salt stress varies with habitat conditions, as N, P, and salinity levels affect both algal survival and development. The authors highlight the importance of considering the potential effect of various environmental factors on salt stress adaptability, particularly cellular proline accumulation.

Park and Lee [24] investigated the relationship between land-use types and water quality indicators at two spatial scales (watershed and riparian) using ordinary least squares and geographically weighted regression models. Their results show water quality indicators to be significantly affected by agricultural and forested areas at both scales, with the watershed scale effective for the management and regulation of watershed land use. Their results reinforce the importance of watershed management in the planning, restoration, and management of stream water quality.

Lee et al. [25] evaluated the mechanistic pathways between land use and the deterioration of both stream water quality and biological assemblages using a structural equation model (SEM), reflecting the impact of agricultural and urban land use on water quality and the benthic macroinvertebrate index (BMI) in river systems. The estimates from the SEM showed that an increase in both urban and agricultural land use percentage directly increased the biochemical oxygen demand (BOD) and TP while decreasing the BMI of streams. The urban and agricultural land use percentage indirectly lowered the BMI through increasing BOD; an increase in nutrients did not lower the BMI. In summary, the results of the study indicate that increased urban and agricultural land use in watersheds both directly and indirectly affects the physicochemical characteristics and biological communities of streams.

Lee et al. [26] analyzed the social value of ecosystem services for resilient riparian greenway planning and management, based on a survey of residents living near an urban riparian greenway. Cluster and importance-performance analyses were used to evaluate the collected data, and based on their results, a Strong Social Value of Ecosystem Services group and a Neutral Social Value of Ecosystem Services group were identified. Different distributions were found between the two groups based on gender and residency period, with significant differences for age and familiarity with the riparian greenway. Results from the study highlight the perceived value of ecosystem services based on group characteristics, and the importance thereof of resilient riparian greenway planning and management approaches.

## 3. Conclusions

Cultural eutrophication is one of the primary challenges to sustainable water management and needs urgent attention due to cumulative anthropogenic activities. It is increasingly recognized that eutrophication has multidimensional consequences linked to water quality, ecosystem and human health, as well as economic activities. Furthermore, the enrichment of nutrients interacts with many site-specific conditions, specifically, the ecological stability of the system, land-use types, climate change, and the presence of other contaminants, including infectious disease agents. It is essential that future studies acknowledge and consider the complexity of these multiple interactions.

## Figures and Tables

**Table 1:** Classification of selected articles published in the Special Issue, Eutrophication and the Ecological Health Risk, Environment Science and Engineering Section, IJERPH, 2020.

Classification	Authors	Theme	Reference
**Eutrophication**	Kong, M.; Chao, J.; Han, W.; Ye, C.; Li, C.-H.; Tian, W.	Degradation characteristics of phosphorus in phytoplankton-derived particulate organic matter	[17]
	Wan, J.; Yuan, X.; Han, L.; Ye, H.; Yang, X.	Characteristics and distribution of organic phosphorus fractions in the surface sediments of the rivers	[18]
	Zulkefli, N.S.; Kim, K.-H.; Hwang, S.-J.	Degradation of extracellular environmental DNA from a eutrophic lake	[19]
**Ecological Health Risk**	Kim, K.; Yoon, Y.; Cho, H.; Hwang, S.-J.	Detecting an odorous compound (2-methylisoborneol) in cyanobacteria	[20]
	Ji, C.W.; Park, Y.-S.; Cui, Y.; Wang, H.; Kwak, I.-S.; Chon, T.-S.	Response behavior of oligochaete to Copper Sulfate	[21]
	Jo, H.; Choi, B.; Park, K.; Kim, W.-S.; Kwak, I.-S.	Gut content analysis of midge larvae (Chironomidae) using a DNA meta-barcoding approach	[22]
	Park, M.-H.; Park, C.-H.; Sim, Y.B.; Hwang, S.-J.	Physiological response of a green alga to salt stress in relation to proline synthesis	[23]
**Ecosystem Health and Service**	Park, S.-R.; Lee, S.-W.	Spatial- and scale-dependent relationships of land-use types with stream water quality	[24]
	Lee, J.-W.; Lee, S.-W.; An, K.-J.; Hwang, S.-J.; Kim, N.-Y.	Effects of land use on water quality and benthic macroinvertebrates in streams	[25]
	Lee, J.; Kweon, B.-S.; Ellis, C.D.; Lee, S.-W.	Value of ecosystem services for resilient riparian greenway planning and management in an urban community	[26]
